# Disease-related data patterns in cerebrospinal fluid diagnostics: medical quality versus analytical quantity

**DOI:** 10.3389/fmolb.2024.1348091

**Published:** 2024-09-11

**Authors:** Hansotto Reiber

**Affiliations:** CSF and Complexity Studies Form, University Goettingen, Goettingen, Germany

**Keywords:** external quality control schemes, cerebrospinal fluid diagnostics, disease-related data patterns, Reiber diagram, blood–brain barrier functions, immunological networks, biophysics in medicine, accreditation system

## Abstract

Cerebrospinal fluid (CSF) diagnostics is characterized by the biologically relevant combination of analytes in order to obtain disease-related data patterns that enable medically relevant interpretations. The necessary change in knowledge bases such as barrier function as a diffusion/CSF flow model and immunological networks of B-cell clones and pleiotropic cytokines is considered. The biophysical and biological principles for data combination are demonstrated using examples from neuroimmunological and dementia diagnostics. In contrast to current developments in clinical chemistry and laboratory medicine, CSF diagnostics is moving away from mega-automated systems with a constantly growing number of individual analyses toward a CSF report that integrates all patient data. Medical training in data sample interpretation in the inter-laboratory test systems (“EQA schemes”) has become increasingly important. However, the results for CSF diagnostics (EQAS from INSTAND) indicate a crucially misguided trend. The separate analysis of CSF and serum in different, non-matched assays and extreme batch variations systematically lead to misinterpretations, which are the responsibility of the test providers. The questionable role of expensive accreditation procedures and the associated false quality expectations are discussed. New concepts that reintegrate the medical expertise of the clinical chemist must be emphasized along with the positive side effect of reducing costs in the healthcare system.

## 1 Introduction

The analysis of cerebrospinal fluid (CSF) for the diagnosis of neurological diseases has always been a particular challenge for clinical chemistry (Reiber, 2016c). The small extraction volume and the low analyte concentrations in the CSF required an improvement in the sensitivity of the analytical methods commonly used in clinical chemistry. The interpretation of the CSF data then became particularly challenging due to the need to differentiate between the fractions in the CSF originating from the blood and those originating from the brain, e.g., immunoglobulins. This led to the combined analysis of the CSF sample with the corresponding blood sample of the patient and the calculation of their ratio as the CSF/serum concentration quotient. This was the beginning of the evaluation concepts of combined data, a discussion that has now lasted 60 years. The linear index, the ratio of the serum protein quotient to the albumin quotient as a reference, remained popular despite the empirically and biophysically based non-linear relationships, e.g., those represented as hyperbolic lines in the quotient diagrams. The progress of neurochemical diagnostics (Wildemann et al., 2010; Reiber, 2016a; 2016b, 2016c) was initially less due to a growing number of new analytes than due to a biologically and medically relevant compilation of data patterns.

The additional introduction of a cumulative CSF data report, which integrated the patient clinical data in 1979 (Reiber, 2016c), became a model for other disciplines of clinical chemistry. A recent tutorial CSF App (Albaum and Reiber, 2024) illustrates this development of disease-related data patterns in CSF diagnostics (Wildemann et al., 2010; Reiber, 2016a; Reiber, 2016b).

The initial development of CSF diagnostics in the 1990s would not have been so successful without the support of Beckman and Dade Behring, the suppliers of the automated nephelometer machines. In the meantime, a decisive role change took place: industrial companies run the analytical invention according to their own rules, which are based more on their financial interests than on medical needs. This means disadvantages for the analytical quality of the combined CSF and serum analysis, as well as an explosion in analytical costs without any corresponding medical benefit. The certification and accreditation business with its high costs also contributes to the loss of quality in clinical neurochemistry as the providers of established online analysis software cannot afford to maintain their service.

### 1.1 Topics of the contribution

1. In CSF diagnostics, the following principles are used that can be generalized to improve the quality of laboratory medicine:
• Quotient formation (CSF/serum proteins and dementia marker proteins).
• Coefficients of variation (CVs) in CSF and blood (brain- and blood-derived proteins).
• Immunoglobulin class patterns with brain-specific preconditions.
• External quality analysis schemes (EQASs) with additional interpretation of medically relevant data patterns.

The current development in clinical neurochemistry is critically discussed with reference to the database of the INSTAND external quality assessment schemes (EQAS) for CSF (Reiber, 1995) and the current certification practice.

2. The integration of disease pathology and current knowledge bases must be part of any quality control of medical laboratory data. The second main aspect of this article is, therefore, the presentation of the medical and biological knowledge bases relevant for the plausibility control and, finally, the correct interpretation of diagnostic data. The obvious deficits in the practice of diagnostic interpretations (Uhr, 2024) and the subsequent lack of adequate therapeutic consequences make this an important goal (Reiber, 2024).

### 1.2 Current knowledge bases

The review of the following topics presents some essentials of clinical neurochemistry in particular and clinical chemistry in general, based on a recent publication (Reiber, 2024):

• The diffusion-flow model of blood–brain barriers
• The immunological networks (B-cell-based and pleiotropic cytokines).
• Biophysics and complexity approach in medical diagnostics.

In times of AI-based big data analysis, the call for a shift from a growing number of individual analytes that lack rational argumentation to a limited amount of functionally linked data seems to be swimming against the tide. However, it is not. In medicine, there are no sufficiently large datasets for disease group statistics with deep learning approaches; conversely, the perspective for AI with machine learning is to integrate an existing secure knowledge base to ensure the reality of categorizations (Wahlster, 2021).

With the integration of current knowledge bases, CSF diagnostics shows the relevance of data coupling to obtain a disease model-based selection of diagnostic datasets. The increasing acceptance of complex system approaches in diagnostics and EQASs in CSF show the foundations of a medical-based quality control that goes beyond the usual external accuracy control of individual analytes.

These aspects could contribute to the perspectives in medical laboratory diagnostics and restore the medical competence of clinical chemists.

## 2 Change of a paradigm: blood–brain and blood–CSF barrier functions

The barrier function is a fundamental topic of this work. A shift from mechanical, linear models to dynamic, non-linear biological functions requires more fundamental knowledge in medicine.

The most glaring example of a necessary paradigm change is the dysfunction of the blood–cerebrospinal fluid barrier, i.e., the pathological increase of serum protein concentrations in the cerebrospinal fluid. The serum protein concentrations in the CSF increase due to a reduced CSF flow rate, i.e., slowed removal, and not due to a hole in the barrier. However, the practice in scientific publications looks significantly different. A Google search under the keyword *blood–brain barrier* with 94,500 citations in the last 10 years is associated 78,900 times with “impairment,” 71,900 times with “breakdown,” and 56,400 times with “leakage” (Google search on 14 March 2023, period 2013–2023).

The idea of a barrier leak is as wrong as the expectation that a stone thrown into the lake will leave a hole in the water.

From an evolutionary point of view alone [see below and Reiber (2024)], such spontaneous instability of a structure that has existed in a variety of species for 500 million years is unlikely.

### 2.1 Barriers

The blood–brain and blood–cerebrospinal fluid barriers are morphologically different and also vary in different brain areas. The molecular passage between blood and extracellular fluid (ECF), the blood–brain barrier, and the passage from blood to CSF, the blood–cerebrospinal fluid barrier, are based on two basic transfer ways, the paracellular passage with facilitated or active transfer mechanisms and the intercellular passage for proteins, which depends on passive diffusion (Reiber, 2024). The biophysical principles in both barriers are the same, but the CSF has a 10-fold faster flow (turnover) than the ECF in the brain.

*Barrier function for proteins*: Serum proteins pass through the endothelial cell layer of the capillaries, which are reinforced with additional brain-specific structures, different in different areas of the brain. The molecular size-dependent restriction of the diffusion of proteins into the CSF is at a steady state with the elimination by CSF outflow (bulk flow). This steady state leads to very low–normal concentration ranges in the CSF, with approximately 1/100–1/3,000 (IgM) of serum concentrations for the most common proteins with the corresponding analytical problems.

### 2.2 Barrier dysfunctions

**FIGURE 1:**
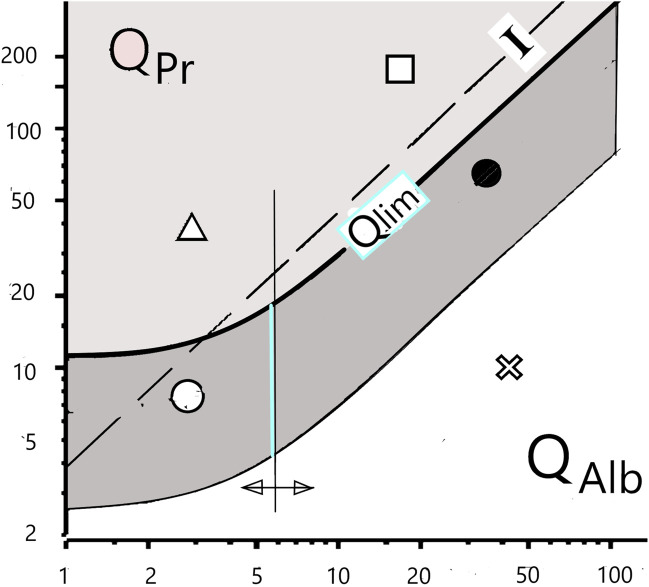
Differentiation of blood and brain fractions of proteins in the cerebrospinal fluid. The hyperbolic borderline (Qlim) is the result of the pathologically altered CSF flow rate, which has a non-linear influence on the local diffusion flow of proteins at the barrier interface to the CSF space. The previous assumption of a linear relationship (“Index I”) is subject to errors of interpretation. The still frequent interpretation of a dysfunction of the blood–cerebrospinal fluid barrier, i.e., an increased QAlb, as a leakage in the barrier structure must be replaced by the new paradigm of a reduced cerebrospinal fluid turnover with an unchanged barrier structure.

In many neurological diseases, the concentrations of serum proteins in CSF are pathologically increased, which is diagnostically characterized by the increased albumin CSF/serum concentration quotient, QAlb (Figure 1). The cause, a pathologically reduced CSF turnover, has three possible sources:

- The inflammation-related disturbance of CSF production in the ventricular plexus;
- A blockage in the subarachnoid space (tumor and stenosis); or
- A reduced outflow of CSF (swelling at the spinal roots in Guillain–Barré syndrome [GBS]).

The new paradigm for the barrier dysfunctions is the biophysically derived and patho-physiologically validated *diffusion-flow model* of the barriers (Reiber, 2003; Reiber, 2021a; Reiber, 2021b). It provides a rationale for the correct interpretation of a barrier dysfunction. Even the age-dependent increase in the normal QAlb value can be explained by reduced CSF production due to age-related changes in the choroid plexus. The increasing QAlb value, the barrier dysfunction, thus represents a reciprocal function of the pathologically decreasing CSF flow rate (Figure 1).

### 2.3 Discrepancies in interpretations

Figure 1 shows a consequence of the changing paradigms. The flow-related change in the local molecular flux, dependent on a non-linear concentration gradient across the barrier (Reiber, 2003; Reiber, 2021a), leads to non-linear, hyperbolic equations of the blood–CSF barrier function instead of linear correlations (Index, I, Figure 1). QAlb is the general reference value for the individual barrier function. It is a gift of nature that albumin, the largest serum protein fraction, is synthesized only in the liver and not also in the brain in pathological processes.

The discrimination between a protein fraction (e.g., IgG) diffusing from the normal blood across the barrier into the brain and a pathological protein fraction synthesized in the brain (intrathecal IgG fraction) is characterized by a hyperbolic limit function either in quotient diagrams (Qlim in Figure 1) or as a mathematical equation (Reiber, 2020). The still frequently assumed linear relationship (Index, I, in Figure 1) leads to interpretation errors. Calculation software for numerical and graphical statistical treatment of CSF data in diseases is available (free software from www.albaum.it), as explained by Reiber (2020).

The example of the blood–brain barrier function shows that without an understanding of the physics of diffusion and the stability of biological structures through material self-organization (see below), an adequate interpretation of the empirical data could be missed.

## 3 Neuroimmunology and immune networks

### 3.1 Immune reactions in the brain

On one hand, this example of a changed knowledge base replaces the clonal selection model of immunity an on the other hand, the idea that the brain is immunologically isolated. The immune system reacts as a network of B-cell clones, and the brain is linked to the immune and endocrine systems by pleiotropic cytokines. This leads to a new interpretation of the development of autoimmune diseases and immune system-associated pathologies such as bipolar spectrum disorders in psychiatry, which miss classical signs of immune reaction in the CSF.

Interpretations of the immune response in brain need to consider the following processes:

• All antibody-forming B cells in the brain migrate from the blood across the barrier into the brain and proliferate in the perivascular lymphocyte cuffs.
• The brain does not have an isotype switch (IgM to IgG class), which only happens in the lymph nodes.
• Antibody maturation (increase in avidity) happens only by selection of B-cell clones in the lymph nodes.
• The specificities of the polyspecific antibodies locally vary due to the random immigration of B cells of different specificities (the Ig class and measles, rubella, and varicella-zoster [MRZ] antibody patterns are different in the CSF and aqueous humor of the same individual).
• Immunocompetent cells of the CNS share cytokine receptors that link the nervous system to the immune and endocrine systems by pleiotropic cytokines.
• The innate immune system produces important components [e.g., of the complement system (Reiber et al., 2012)] in the brain, but their functions in disease development and defense (Jack et al., 2001) are not well understood.
• The polyspecific nature of any systemic and intrathecal immune response is the base for the diagnostics of chronic immune system-associated diseases (Reiber, 2024)

### 3.2 Immunological networks

The idea of the immunological network dates back to 1975, with numerous theoretical approaches being developed in the 1980s (Varela and Coutinho, 1989; De Boer and Perelson, 1991). These theories extended the classical view that the adaptive immune system reacts to invading substances by producing antigen-specific antibodies (clonal selection) to rid the body of invaders. With the discovery of anti-idiotypic antibodies that recognize the body’s own antibodies, lymphocyte-based networks became relevant. With the discovery of the polyspecific nature of the immune response in CSF, CSF research has made a major contribution to the practical significance of these theories. Felgenhauer et al. (1985) first described measles virus antibodies in the CSF of MS patients. Meanwhile, the observation that all immune responses, whether acute or chronic, involve polyspecific antibody synthesis (Reiber et al., 1998; Reiber, 2017a) has made the network property of the immune response an obligatory knowledge base for the interpretation of antibody data. In addition to this B-cell network, the trans-organ cytokine network associated with immunocompetent brain cells has also become important for the understanding and diagnosis of diseases related to the immune system, especially chronic diseases (Reiber, 2017a; Reiber, 2017b; Reiber, 2024).

#### 3.2.1 Specific and polyspecific antibody synthesis in brain

The combined intrathecal MRZ antibody reaction in multiple sclerosis (Reiber et al., 1998), which is now of diagnostic relevance due to its strikingly high frequencies (Table 1), has also been reported in other chronic diseases (Hottenrott et al., 2015).

**TABLE 1:** Polyspecific antibody synthesis in the CNS in multiple sclerosis. Mean frequencies of intrathecally synthesized antibodies in multiple sclerosis patients against measles (M), rubella (R), varicella zoster (Z), herpes simplex (H), chlamydia (Chl), human herpes virus 6 (HHV6), toxoplasmosis (Tox), *Borrelia* (Bo), and double-stranded DNA (ds D). The mean values depend crucially on the respective MS cohort, depending on the intensity of intrathecal synthesis (Reiber et al., 1998). The patterns of polyspecific antibodies in the CSF vary from patient to patient.

Ab-species	M	R	Z	H	Chl	HHV6	Tox	Bo	ds D
Frequency in MS (%)	78	60	55	28	30	20	10	<25	19

A fundamental new understanding came with the discovery in CSF diagnostics that in the case of an immune reaction to a specific antigen, as in herpes encephalitis (HSV) and subacute sclerosing panencephalitis SSPE (measles) (Jacobi et al., 2007), the disease-related specific antibodies account for only a small proportion of the total intrathecal antibody synthesis (Table 2). Nevertheless, the causative, specific antibodies have a 40–60-fold higher intrathecal synthesis rate than the polyspecific antibodies in chronic diseases (Jacobi et al., 2007).

**TABLE 2:** Comparison of intrathecal antibody responses to specific and polyspecific immune reactions. Calculated as specific intrathecal fraction Fs and expressed as the percentage of specific antibodies in relation to total intrathecally synthesized IgG (Jacobi et al., 2007). Measles-Ab and HSV-Ab are CSF values in subacute sclerosing panencephalitis, herpes simplex encephalitis, and multiple sclerosis. Rubella-Ab was determined in the aqueous humor (AH) of the eye in the Fuchs heterochromic cyclitis (FHC) and uveitis/periphlebitis of the eye as an MS symptom, MS(U).

Antigen	Specific Ab (%)		Polyspecific Ab (%)	
Measles (CSF)	18,8	SSPE	0,52	MS
Herpes s. (CSF)	8,9	HSVE	0,14	MS
Rubella (AH)	2,6	FHC	0,06	MS (U)

In addition, the polyspecific antibodies differ from the specific antibodies by their generally higher avidity (Gharavi et al., 1996) (Table 2) as a consequence of the longer time interval for antibody maturation available in the case of chronic diseases.

#### 3.2.2 Connectivity of B-cell clones in blood

The polyspecific immune reaction as a general feature of the immune response can also be detected in the blood. A representative example comes from a brilliant study of GBS patients (Terryberry et al., 1995) with individually randomly elevated antibody and autoantibody titers in the blood. The pattern of elevated titers of polyspecific antibodies (Ab) is individually variable in an analysis set of 22 antibody assays in the 56 GBS patients (Terryberry et al. 1995), with between 1 and 13 simultaneously elevated titers, suggesting a Gaussian distribution for the arbitrary test set when the data are re-evaluated (Reiber, 2017a). The true network depth [connectivity (De Boer and Perelson, 1991)] in the individual patient, which includes autoantibodies, cannot be determined from the analyzed, arbitrary, and very limited test set.

The connectivity of B-cell clones was also demonstrated in a direct dynamics study.

**FIGURE 2:**
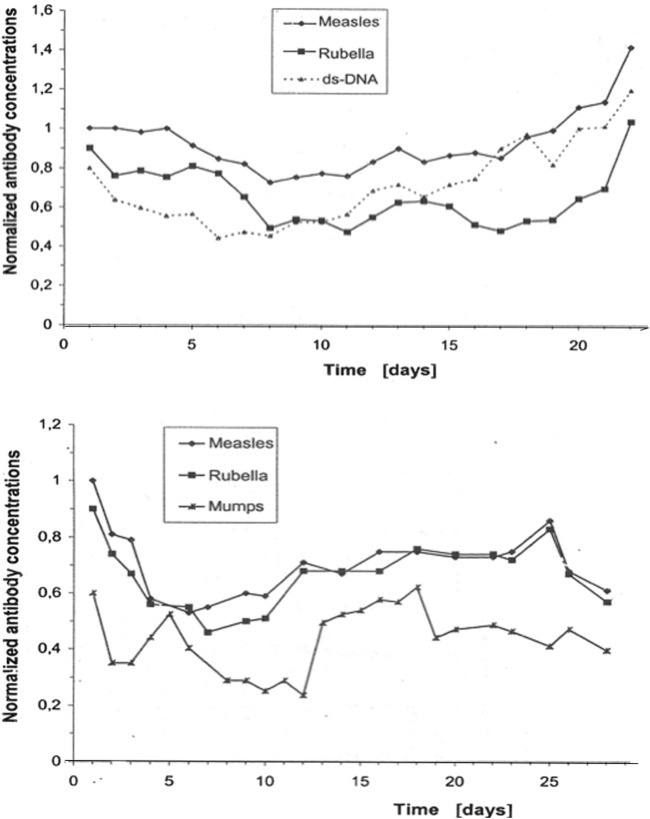
Antibody dynamics in the blood of patients without inflammation. The data originate from daily routine blood samples in the neurological intensive care unit of patients with a non-inflammatory disease (stroke) (Heitmann, 2002). These concentrations correspond to normal titers, i.e., they are below the threshold for nosocomial infections. The collected frozen samples were measured together in one analytical run (microtiter plates) to avoid bias due to inaccuracies in day to day imprecision (intra-assay imprecision CV = 3.5%). The concentrations of the individual antibody species were normalized to the first analytical value with C = 1. The total fluctuations in the blood proteins were controlled to exclude blood volume fluctuations as a cause. The curves are shifted slightly parallel along the *Y*-axis to better visualize the coupled daily concentration fluctuations.

The correlation of daily fluctuations in antibody concentrations in the blood of control subjects (Figure 2) also shows that connectivity is a feature of any normal immune response (Heitmann, 2002). Some Ab species fluctuate in a coupled manner, and others, independently. In both patients shown in Figure 2, the fluctuations in measles and rubella antibody concentrations are correlated with each other but not with dsDNA autoantibodies or mumps antibodies.

In addition to the specific antibodies, every acute infection with a specific antigen also leads to increased polyspecific antibody synthesis by a large number of other, already existing B-cell clones against other microorganisms, even autoantigens.

### 3.3 Connectivity-based interpretations—chronic immune reactions

Connectivity in the immune system explains many frequently misinterpreted observations. Only through the polyspecific activation of different coupled B-cell clones can lifelong immunity be maintained, as observed with measles immunization. The short lifespan of the B cells would not allow this if the specific B-cell clones were not maintained in the system through constant polyspecific activation.

The individual network depth of the connected B-cell clones determines the duration and efficiency of immunization (wild-type infection or vaccination). Connectivity can also be the cause of an autoimmune reaction by activating an existing B-cell clone for autoantibodies. When low levels of autoantibodies that provide immune tolerance are upregulated by a factor of 50, an autoimmune response appears as an undesirable damage associated with the individual immune response.

The Danish Disease Register (Nielsen et al., 2016) shows that autoimmune diseases and symptoms such as chronic fatigue syndrome/ME can occur a few weeks after the occurrence of infectious diseases. This also corresponds to post-Lyme or post-COVID symptoms. These correlations of non-specific, delayed immune reactions also make it clear that vaccinations can act as polyspecific triggers and, thus, also make the largely suppressed connection of the Gulf War illness with the 8-fold vaccinations in the first Gulf War plausible (Reiber and Davey, 1996; Rook and Zumla, 1997; Nielsen et al., 2016; Reiber, 2017b). Post-COVID syndrome has also been observed after vaccination, albeit less frequently than after wild-type infections. These associations offer a new approach to chronic diseases associated with the immune system that were previously difficult to diagnose (Reiber, 2024).

### 3.4 Cytokines, the trans-organ network

In the brain, cytokines are produced in immunocompetent cells, astrocytes, microglia, and endothelial cells. They can have both pro-inflammatory and anti-inflammatory effects. This is an important aspect of the immune response in the brain as a self-organizing local process, which is thought to be involved in various psychiatric chronic diseases (Bechter et al., 2010; Bechter, 2020; Runge et al., 2021).

The network is formed by the three properties of the different cytokines:

- Functional pleiotropism.
- Functional redundancy.
- Up and downregulation.

Pleiotropism refers to the binding of a cytokine to receptors in different organ systems. This creates a link between the three central control systems of the organism: nervous system, endocrine (hormonal) system, and immune system. Functional redundancy means that different cytokines can have the same effect in one and the same organ.

Since one and the same cytokine can also be upregulating and downregulating depending on its concentration, a very complex network is created that is influenced by different body systems. This explains experiences that have led to the establishment of research concepts such as psychoneuroimmunology and diseases such as “stress-induced para-inflammation” (Bechter, 2020).

The involvement of cytokines in the initial processes of brain inflammation via the choroid plexus (Castellani et al., 2023) is also fundamental information for understanding the frequent barrier dysfunction in neurological diseases as a consequence of reduced cerebrospinal fluid production in the plexus.

The inclusion of cytokine analysis must become a fundamental requirement in neurology and psychiatry in order to explain pathologies associated with the immune system.

## 4 Biophysics in medicine

### 4.1 Complexity and Selforganization of Matter

The above examples of barrier function and the immunological network have highlighted a deficit in medical development. This becomes even clearer when we consider the unsuccessful therapeutic research into chronic diseases. There is no chronic disease for which we have a causal therapy, be it high blood pressure, type 2 diabetes, multiple sclerosis, autoimmune diseases, post-infectious chronic fatigue syndrome, heart failures, or the many types of cancer. This is primarily the result of the lack of complexity of the predominantly linear disease models, which are based on simple cause–effect relationships.

Fifty years ago, glycolysis oscillation (Hess and Boiteux, 1980; Gerok et al., 1989; Reiber, 2024) described how even in the smallest metabolic process of the cell, oscillating regulatory states can spontaneously change from one rhythm to another due to non-linear functions. This phenomenon is explained by complexity science as a change between attractors.

In addition to obtaining a better understanding of complex systems (Gerok et al., 1989; Goodwin, 2001; Reiber, 2007), we need to move from Jacob and Monod’s refuted, 70-year-old model of the “genetic program” to the relevance of a phenotypic biology with a new view of epigenesis (Reiber, 2012). This also includes a fundamental recognition of the concepts of material self-organization in the context of non-equilibrium thermodynamics (Prigogine, 1997) or the non-linearity of biological processes in the context of complexity science (Mandelbrot, 1991). A short survey on these topics (Reiber, 2024) was published recently, providing more details.

There is no doubt that the improvement in medical diagnostics and therapy requires better biophysical disease models.

### 4.2 Dynamics of analyte concentrations

The daily fluctuations in the concentration of antibodies in the patient blood are shown in Figure 2. The coordinated variations indicate a common complex regulation. In patients with osteoporosis (Gerok et al., 1989), a disorder of calcium/parathyroid hormone regulation, the mean blood concentrations of calcium or parathyroid hormone remained unchanged compared to that in controls. Rather, the transitions changed from a normal, deterministic–chaotic time series of parathyroid hormone concentration in the blood to an almost constant concentration curve, indicating the loss of regulation. Analyzing a single blood sample may be useless for diagnosis, but a time series could provide it.

In contrast to analyzing individual samples, the electrocardiogram in heart disease provides a sufficiently large dataset to create time series (tachograms). The normally chaotic rhythm of the heartbeat (Reiber, 2024) changes under certain conditions to a time series, which can be analyzed for a lower complexity (change in fractal dimension) using complexity science methods (Mandelbrot, 1991; Reiber, 2024).

In general, time series (e.g., from electrocardiograms or electroencephalograms) are not available for clinical chemical diagnostics. However, these theoretical principles show that chronic diseases, in particular, can represent a stable state, i.e., have an attractor. This has consequences for diagnostics and therapy (Reiber, 2024).

This becomes clear, for example, in post-Lyme disease (Reiber et al., 2013): antibody findings must be interpreted differently, and treatment with antibiotics is pointless. The current discussion about post-COVID shares these problems.

CSF diagnostics cannot refer to the serial examination possible in blood, as a CSF puncture is rarely repeated unless for special reasons. This leads to a further concept for the integration of the complexity approach. The identification of disease-related data patterns can compensate, to a certain extent, for the complexity in the processes, as represented in the time series.

## 5 CSF diagnostics benefit from disease-related data patterns

Both the practical and theoretical principles of CSF diagnostics are documented in detail in various references (Reiber, 2016c; Lejon et al., 2003; Jacobi et al., 2007; Reiber et al., 2009; Wildemann et al., 2010; Reiber et al., 2013; Reiber, 2016a; Reiber, 2016b; Albaum and Reiber, 2024; Lewczuk et al., 2021; Reiber, 2024; DGLN 2024). Particular emphasis is placed here on the diagnostic principles that can be generalized for general quality improvement in laboratory medicine by identifying rational data combinations:

• Quotient formation and data variability (CV).
• Intrathecal immunoglobulin class patterns.
• Extended EQAS with the interpretation of data patterns.

### 5.1 Quotient formation—the role of biological variation

#### 5.1.1 Definition and function

The CSF/serum concentration quotient (QAlb, QIgG, etc.) represents a normalized dimensionless CSF concentration that is independent of the individual variation in serum concentration [values between 0 and 1, which are used in practice due to the low CSF concentration in parts per thousand (× 10^-3^)].

This quotient is a biological relationship related to the laws of diffusion and not an arbitrary relationship such as in the calculation of protein concentrations from densitograms of serum electrophoresis. The CSF/serum quotient for proteins from the blood in the CSF excludes interpretation errors caused by greatly altered serum levels. For example, in patients with a serum IgM concentration increased up to 10-fold due to trypanosome infestation (Table 3) (Lejon et al., 2003), a subsequently increased CSF IgM concentration would be incorrectly interpreted as intrathecal IgM synthesis due to the high absolute values in the CSF. As the quotients are only determined by molecular size-dependent diffusion, the quotient remains normal despite extremely high serum concentrations (Table 3), provided that no barrier dysfunction or intrathecal synthesis contributes additional IgM.

**TABLE 3:** Blood concentrations and CSF/serum quotients in trypanosomiasis (stage 1 without brain involvement, parasitosis). Due to the constantly changing surface antigens of the trypanosomes, a new infection is always simulated with a corresponding new IgM class reaction. A 10-fold increased IgM concentration may contribute to the extremely low albumin protein production in the serum.

Proteins	Blood concentrations (g/L)		Mean quotients (x 10^3^)	
	Normal control	Trypanos. 1. stage	Normal control	Trypanos. 1. stage
IgM	0.6–2.5	9–18	0,3	0,3
Albumin	35–55	23,2–33,6	5	5

The CSF/serum concentration quotient has the quality of a method-independent reference value, provided that the CSF and serum values are correctly analyzed in parallel (see below), i.e., the basic rule is no CSF analysis without serum analysis. The diagnostic uncertainty is hidden in the absolute values, not in the quotient calculated from biologically correlated data.

#### 5.1.2 Coefficient of variation for the detection of data connections

For physically or biologically coupled values such as the CSF and serum concentrations of serum proteins (IgG, prothrombin, etc.; Table 4), the CV value of the quotient decreases compared to the absolute individual CSF CVs. The nominator of the quotient (CSF concentration) depends on the molecular size-dependent diffusion from a variable serum concentration (denominator of the quotient) and the individually varying length of the diffusion pathway and the CSF flow rate. Without the latter parameters, we would have a universal constant that correlates with the diffusion coefficient D. The albumin quotient, which is freed from the variability of the serum concentration, still varies with the individually varying length of the diffusion pathway and the CSF flow rate. If, for example, we relate the IgG quotient to the QAlb, we remove these two fluctuations and obtain the universal non-linear hyperbolic relationship between QIgG and QAlb.

**TABLE 4:** Inter-individual coefficients of variation, CVs, of CSF proteins from different sources. CSF, serum, and the CSF/serum quotients of each parameter are always from the same group with serum and CSF analyzed in the same analytical run. Comparison of blood-derived molecules [albumin, IgG, prothrombin (Reiber, 1995)], brain-derived neopterin (Kuehne et al., 2013), predominantly brain-derived proteins [transthyretin (Reiber, 2003)], and the leptomeningeal mannan-binding lectin (Reiber et al., 2012) or beta-trace protein (Reiber, 2003).

	Coefficients of variation, CV (%)						
	Alb	IgG	Prothr	Neopt	MBL	TT	ßTrace
CSF	29.3^1)^	46	33	10	66	6.4	24
Ser	8.9	35	21	25	146	18	28
Q x10^3^	26.2	18	22^2)^	21	81	23	25
N	53	23	18	26	13	28	28

^a^ Limited age interval 50–59 years, mean 55 ± 3.2 years.

^b^ Calculated from Qpr = 3 ± 0.7 at QAlb = 4.5 (Reiber, 1995).

However, for independent parameters such as CSF and serum concentration of a brain-derived molecule (neopterin, MBL, and TT; Table 4), the CV of the quotient increases up to the sum of the two individual CVs. This contradicts the use of a quotient for data interpretation in the cases of brain-derived proteins.

#### 5.1.3 Quotients in dementia diagnostics

The difference between biologically associated (A) and non-associated (B) analytes in process dynamics can be illustrated using the example of dementia diagnostics.

A. The beta-amyloid (1-42)/(1-40) ratio (=quotient).

The decrease in the beta-amyloid (1-42) concentration in the cerebrospinal fluid of a patient correlates with the formation of Alzheimer’s plaques, with exceptions in the early phase. With the invention of the beta-amyloid (1-42)/(1-40) ratio, the inter-individual variations in total amyloid levels could be eliminated by referring to its main isoform, i.e., Aß40. This well-established ratio, which has recently been substantiated theoretically (Lewczuk et al., 2021), improved the diagnostic sensitivity compared to the determination of the beta-amyloid (1-42) concentration alone. This example is also mathematically a normalization that results in a relative change in beta-amyloid concentration corrected for individual differences in total amyloid levels. This has been empirically validated (Lewczuk et al., 2021) using patient groups and controls and shows that in the control group the 42/40 ratio has a 3-fold lower CV than the absolute values for Aß42. With a stronger decrease in the Aß42 concentration, the CV approaches both parameters [Aß42 ≈ Aß(42/40)], but it shows no inversion. In the critical range of the early Aß42 decrease, the ratio has the highest discrimination accuracy, i.e., sensitivity for the early detection of a pathological process.

B. Aß (1-42)/pTau protein ratio

Both proteins express Alzheimer’s disease in different ways, but they provide different diagnostic information. Aß is reduced as an early expression of amyloidosis, the formation of amyloid oligomers, and the accumulation of amyloid-ß plaques. The Tau protein or pTau is increased later as an expression of the degenerative process on the neurons. This has two consequences: 1) in the same patient group, the CV of the ratio is greater than the individual CV of only Aß(1-42), i.e., the ratio is less sensitive, and 2) the ratio between the two analytes is variable depending on the course of the disease and is, therefore, susceptible to misinterpretation, e.g., if the individual patient happens to have a still normal but very low Tau or pTau protein concentration at the time of early Aß decrease. This would lead to a false negative interpretation (normal instead of elevated Aß42 levels). Despite the low sensitivity, however, this association can contribute to diagnostic specificity if the diagnostic question regarding the time of disease progression is framed correctly.

##### 5.1.3.1 Summary

We described three versions of useful ratios between variables in the organism that improve analytical accuracy, diagnostic sensitivity, and diagnostic specificity:

• Biophysical coupling/normalization: decreased, constant CV.
• Biological coupling/normalization: decreased, not constant CV.
• Medical relevant pattern/association: increased CV

These differences need to be understood by regulatory bodies to finally end the useless discussions about the relevance of CSF/serum concentration ratios as part of guidelines as the ratio has less CV variation compared to absolute concentration values.

#### 5.1.4 Research projects: source detection of proteins in CSF

Conversely, the data combination given in Table 4 could be used in research to characterize the origin of a new molecule in CSF based on its CV (Reiber, 2003; Kuehne et al., 2013). Further parameters for characterizing the origin of a molecule in CSF are the gradient depending on the molecular size, the CSF/serum concentration quotient, and the rostro-caudal concentration gradient, which increases in the case of blood-derived or leptomeningeal proteins (Reiber, 2003; Reiber, 2021b).

### 5.2 Immunoglobulin class patterns in neurological diseases

**FIGURE 3:**
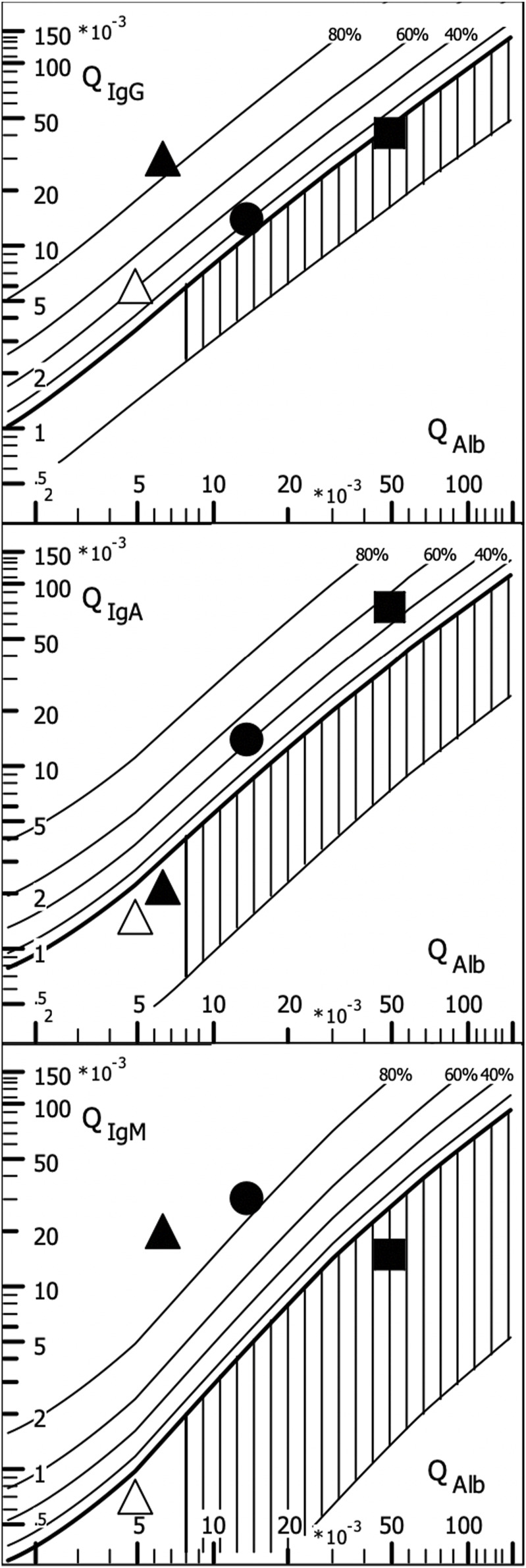
Quotient diagram for IgG, IgA, and IgM (Reibergram). Presentations of patient data for neurotuberculosis (squares), neurotrypanosomiasis (circles), and two patients with neurosyphilis with different courses (triangles). Neurotuberculosis shows a characteristic picture with isolated intrathecal IgA synthesis, whereas in neurosyphilis, conversely, a lack of IgA class reaction is more typical. Neurosyphilis shows a different pattern in the meningovascular course (open triangle) than in the parenchymatous course (filled triangle), with an additional strong IgM class reaction. In African trypanosomiasis, the dominant IgM class reaction with a frequent three-class reaction offers the highest specificity for brain involvement (sleeping sickness). For numerical characterization, the relative intrathecal fractions are used, such as IgG (IF) = [QIgG -Qlim (IgG)]/QIgG × 100 (%) (corresponding % lines in Figure 3).

The disease-typical reaction patterns of intrathecal IgG, IgA, and IgM syntheses differ from the uniform immunoglobulin dynamics in the blood due to the lack of an isotype switch in the brain and the local character of the intrathecal immune response with a barrier passage of the immune cells originating from the blood. By calculating the relative intrathecal fractions of the total immunoglobulin in the CSF (intrathecal fractions, IF, shown as % lines of intrathecal Ig in Figure 3), disease-typical combinations of IgG, IgA, and IgM syntheses can be described in the quotient diagrams, which are also known as Reibergrams or Reiber diagrams (Figure 3).

#### 5.2.1 Representative examples in quotient diagrams

The examples given in Figure 3 show neurotuberculosis, African trypanosomiasis, and two different courses of neurosyphilis:

1. Intrathecal IgA synthesis (Figure 3) in combination with increased CSF lactate, a barrier disorder, and a mean cell count are so highly specific that in European contexts, this is an important clue to an otherwise unexpectedly rare tuberculosis. This pattern eliminates the need to search for other pathogens, contributing to rapid diagnosis and treatment at a minimized cost.
2. The three-class reaction with dominant IgM response in trypanosomiasis (Lejon et al., 2003) (Figure 3) would also fit neuroborreliosis (Reiber et al 2013). However, confusion is impossible if the geographical differences are taken into account and the obligatory detection of trypanosomes in the blood with an extreme serum IgM concentration in trypanosomiasis is added (Table 3).
3. The different course of meningovascular and parenchymal neurosyphilis is shown in Figure 3 by an extremely strong additional intrathecal IgM synthesis (85% of the total IgM in the CSF). The intrathecal IgG synthesis with increased *Treponema* AI in neurosyphilis is found as a scar over decades despite adequate treatment and should not be interpreted as a sign of activity. Reactivation of neurosyphilis can only be recognized by the elevated serum IgM levelsynthesis as neurotuberculosis. Examples.

Further examples are described with interpretations and comments in the CSF Tutor App (Albaum and Reiber, 2024), the reviews (Reiber, 2016c; Wildemann et al., 2010; Reiber, 2016a), and individual publications (Lejon et al., 2003; Reiber et al., 2009; Bechter et al., 2010; Kuehne et al., 2013; Reiber et al., 2013).

In general, this combined analysis of IgG, IgA, and IgM data can provide a number of important diagnostic clues, including the detection of analytical errors through plausibility checks (antigen excess in IgA analysis and blood contamination in CSF).

These associations of disease-related data also show that quality control via medical plausibility must be understood as an important complementary control to EQAS (see below).

#### 5.2.2 Diagnostic sensitivity and specificity of quotient diagrams

The disease-specific Ig patterns have different sensitivities for different diseases and only gain their specificity with additional information. The disease-typical patterns refer to the first diagnostic puncture, which takes place at different times after the onset of the disease, depending on the disease, i.e., 1–2 days for bacterial meningitis, 1 week for viral encephalitis, or up to 3 weeks for neurotuberculosis, depending on the onset of symptoms.

The typical pattern and its sensitivity depend on the course of the immune response in the blood, the variable time for transmission of the microorganism through the barrier, and the locally variable onset of the disease in the brain. It is important to understand that an undetectable immune response in the CSF does not rule out disease as pathological processes far from the CSF space may be undetectable due to the long diffusion pathway to the CSF space. The carcinoembryonic antigen (CEA) is not detectable in the CSF if the CEA-producing tumor metastasis is localized in the frontal brain. This different localization of the pathological processes in the brain is one of the reasons that limits the detection sensitivity of the patterns.

The additional information in the quotient diagram pattern is an essential part of not only the specificity of this diagnostic approach, such as cell count, differential cell count, and lactate, but also the communication of the differential diagnostic questions. This integrating CSF data report [examples in the study by Reiber (2016c) and Reiber (2016a)], introduced in Göttingen in 1977 by the neurologists Sigrid Poser and Helmut Bauer, has become the standard for many laboratories.

However, the interpretation of the patterns undoubtedly requires training and knowledge to avoid diagnostic errors. A CSF Tutor app for the interpretation of disease-related patterns is available as free software (Albaum and Reiber, 2024). This also led to the integration of pattern interpretation into the external quality control systems for laboratory diagnostics as early as 1995 (Albaum and Reiber, 2024).

Disease-related data patterns should be considered as a perspective for analytical plausibility control in order to improve the specificity of diagnostics from a medical point of view, additionally increase the accuracy of interpretation, and, above all, reduce healthcare costs.

## 6 Quality control in the CSF laboratory

Details of quality control in the CSF laboratory have been reported by Wildemann et al. (2010) and in the collection of methods from the German CSF society (DGLN, 2024). These knowledge-based concepts were taken over only to a very restricted extent by the official quality control authorities (RiliBÄK). The improved EQAS of INSTAND (Albaum and Reiber, 2024) was a normative example, which is still the leading, albeit most demanding concept.

### 6.1 Medical data as part of quality control

The evaluation of combined data patterns as part of an EQAS with INSTAND had been introduced in the 1990s (Albaum and Reiber, 2024). In many surveys, we demonstrated that the quality of medical diagnostics does not depend on the smallest possible coefficient of variation of a method but on the interpretability of laboratory data by the clinical chemist and, finally, the physician. The Göttingen neurologist Klaus Felgenhauer, who emphasized the importance of laboratory analyses, said, “If a laboratory finding does not fit into my clinical diagnostics, I ignore it.” This can save lives, but it can also cause an alternative diagnosis to be missed. This aphorism became reality when a laboratory report of an isolated intrathecal IgM synthesis with a normal cell count and other normal protein concentrations was ignored as the clinicians had discarded an inflammatory process. So, the alternative interpretation as a non-Hodgkin lymphoma in the CNS was missed by the neurologists, and it needed a particular case conference to find the diagnosis with delay. To query these options of intrathecal IgM synthesis in an external quality control system contributes more to the quality of laboratory diagnostics than the correctness of absolute IgM concentration values. Some control institutions (Controllab, Brazil) cover this aspect of knowledge-based interpretations by an occasional question/answer test. INSTAND is hosting an online conference of the surveyor with the customers, in addition to the explicit medically oriented commentary, with the results reported to the participants.

### 6.2 Misleading industrial trends in clinical chemistry

#### 6.2.1 CSF protein analysis

The ideal method is to measure the CSF and correspondingly diluted serum in the same assay with a calibration curve that is true to dilution (same recovery in all concentration ranges). Thankfully, this concept was originally developed by Beckman and Dade Behring for their nephelometer machines in cooperation with the Neurochemical Laboratory in Göttingen (head: H. Reiber). The immunochemical analysis with antigen-coated microtiter plates served as the reference method, which is by far the most sensitive method. In the meantime, other suppliers of test systems for basic CSF protein analysis participate in the survey (Siemens, Roche (turbidimetry), Beckman, Abbott, Binding Site, and others).

#### 6.2.2 Actual analytical quality problems

The current, very serious analytical problems are demonstrated in several latest surveys (Uhr, 2022), with interpretations and comments by Manfred Uhr, the current surveyor of the CSF inter-laboratory test at INSTAND (Uhr, 2022). The following results focus on the performance of participants using Siemens analyzers, which form the largest group and also have the most problematic performance due to the conceptual decisions of the assay provider. There are three main problems.

1. Separate analysis of the CSF and serum samples of a patient in different assays.
2. Non-matching calibration curves in the CSF and serum assays (bad recovery).
3. Large variations from batch to batch in the CSF assay.

##### 6.2.2.1 Unreliable recovery in unmatched CSF and serum assays

**FIGURE 4:**
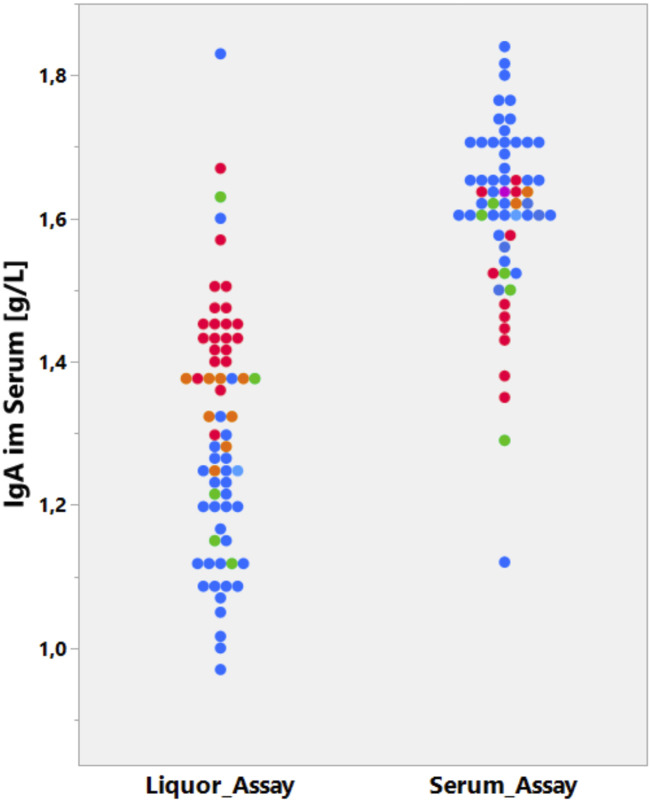
Data from the CSF EQAS of INSTAND, May 2022 (Uhr 2022). Method-dependent accuracy. Analysis of serum IgA in CSF or serum assay. Absolute IgA concentrations in serum samples are determined either in a serum assay (right column) or diluted in a CSF assay (left column). The CSF and serum assays that are from the same supplier (Siemens, blue) are not matched and must lead to a wrong CSF/serum IgA quotient.

The problems start at the lowest level: assay manufacturers avoid the cost of matching the calibration of their CSF assay with the calibration of the serum assay to ensure the accuracy of the CSF/serum quotient (e.g., CSF/serum concentration quotient, QIgG). This is shown in Figure 4.

Figure 4 summarizes the serum IgA concentration measured by one group of participants with the serum IgA assay and (the same serum sample diluted) the CSF assay by another group (Uhr, 2022). The strongest difference in the recovery of the serum IgA concentration between CSF and serum assays was observed in participants using the Siemens assays (blue).

##### 6.2.2.2 Misinterpretation due to unmatched CSF and serum assays

**FIGURE 5:**
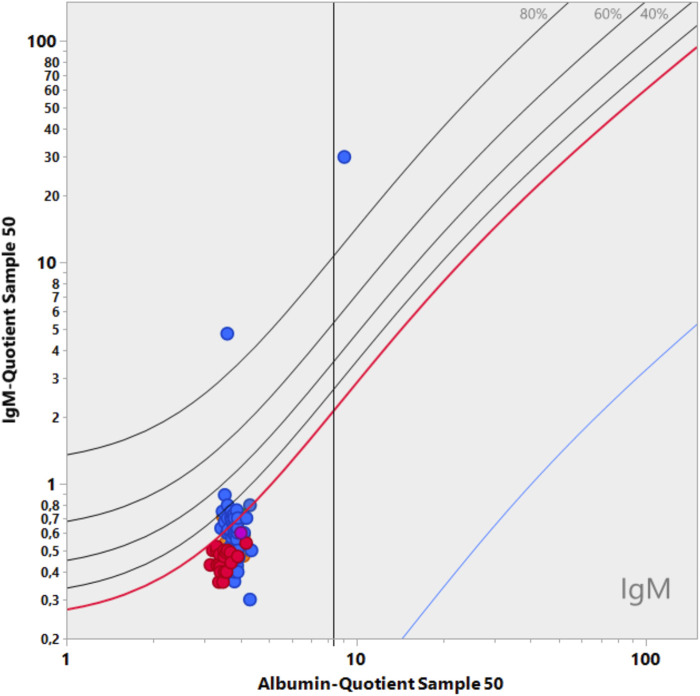
Data from the CSF EQAS of INSTAND, September 2022 (Uhr, 2022). Interpretation error in quotient diagrams. The absolute serum IgM values were analyzed in either the serum or CSF assay. If CSF and serum (diluted) were measured in one group in the same CSF assay, the CSF/serum IgM quotient led to a correct normal quotient, which is below Qlim in the diagram. The group that measured CSF in the CSF assay and serum in the serum assay yielded a wrong interpretation, indicating an intrathecal synthesis (QIgM greater than QIgMlim). Both assays were from the same supplier (Siemens).

Figure 5 shows the fatal consequences of IgM serum analysis with unmatched assays for a CSF/serum sample pair. Participants with the Siemens assay who measured the serum with the serum assay received a quotient that was too high, which indicated a pathological IgM value (QIgM > QIgMLim). As IgA and IgG levels were normal in the patient, the result is misinterpreted as an inflammatory process or can even be interpreted as an indication of intrathecal lymphoma due to the isolated intrathecal IgM synthesis. In daily practice, this would lead to a serious health risk for patients and an extreme increase in costs in the clinic (see example above).

##### 6.2.2.3 Inaccuracy from batch to batch

**FIGURE 6:**
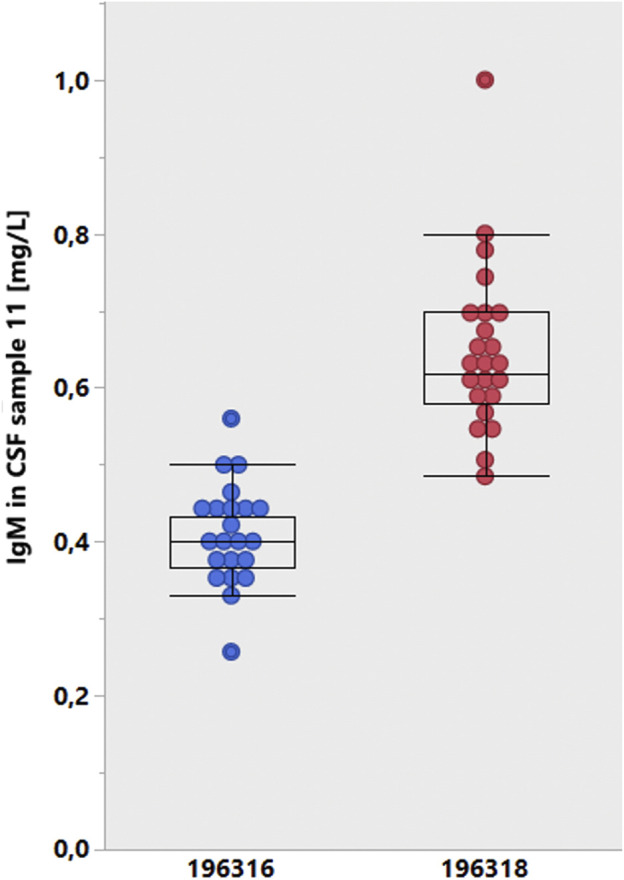
Lot-dependent test results of the INSTAND survey (Uhr, 2022, January 2020). The results were cumulated from participant groups according to the lot numbers of the Siemens Healthcare tests for IgM in CSF. A mean lot-to-lot difference of 50% is hardly reliable.

The second bias by the test provider results from the inadequate difference between batches of CSF tests (Figure 6). When analyzing the CSF samples with different batches from the provider (Siemens Healthineers), a difference of 50% was found between the two groups (Figure 6), which represents a problem of quotient accuracy, especially when the CSF and serum analyses are performed with different assays. Siemens made a conscious decision by canceling the certificate of the CSF assays for serum analysis. This manufacturer-dependent performance can lead to fatal consequences for the patient, as the interpretation example in Figure 5 shows.

**FIGURE 7:**
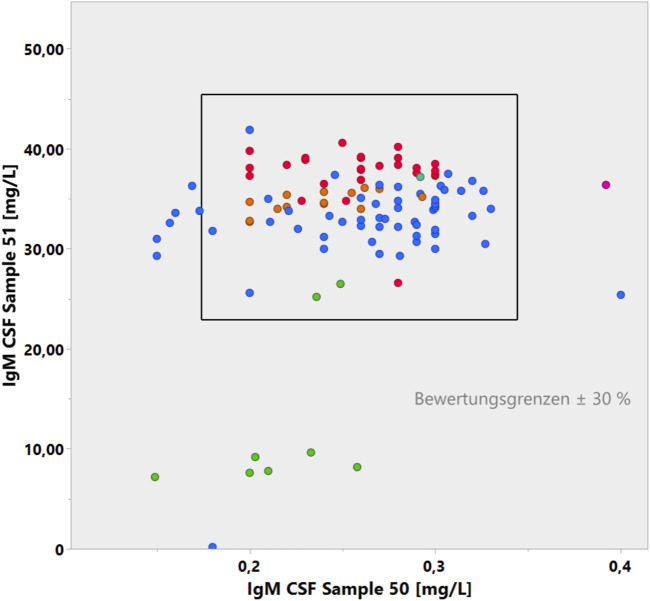
Youden plot of IgM concentrations in two CSF samples in the CSF EQAS (Uhr, 2022, September 2022). The accuracy of the lower concentrations in sample 50 with a larger coefficient of variation does not match the accuracy of sample 51.

Given the bias in analyzing CSF and serum samples in different assays (Figures 4, 5) and the amplifying bias due to different accuracies in different CSF assay batches (Figure 6), it would be reasonable to insist in principle that CSF and serum samples for protein analysis are measured in the same assay with reference to the same calibration curve and that the dilution accuracy of the common calibration curve is guaranteed, which is a basic problem, as shown in Figure 7.

### 6.3 Knowledge-based pattern interpretation as part of EQA systems

#### 6.3.1 Structure of the survey

**FIGURE 8:**
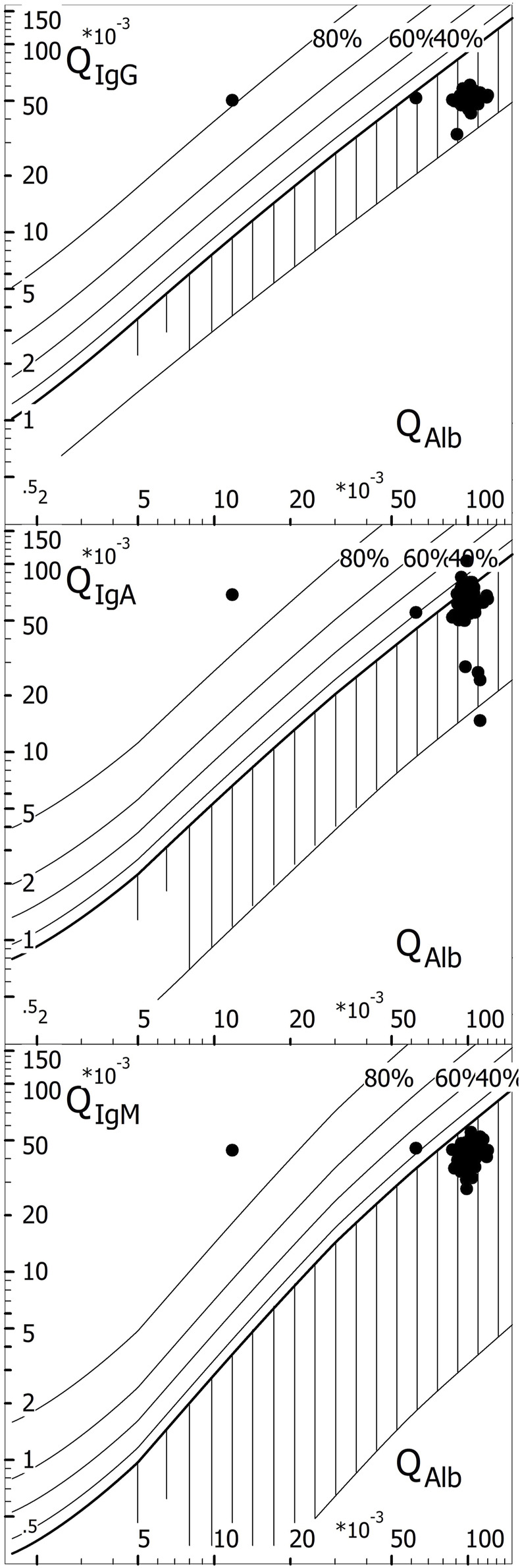
Knowledge-based interpretation of borderline intrathecal IgA synthesis as neurotuberculosis. Example in the EQAS (Uhr, 2022, survey 2021, October). The IgA quotient of 64.6 × 10^-3^ was greater than the IgG quotient of 51.6 × 10^-3^ (confirmed by 124/125 participants). Only 33% of all participants with a complete analysis (42/125) reported intrathecal IgA synthesis with interpretation as an inflammatory process. N = 12 participants calculated QIgA > QIgA (lim). Of the 113 participants with values < Qlim, only 30 (27%) recognized that if QIgA > QIgG, the interpretation must be intrathecal IgA synthesis. Thus, 67% of all participants who analyzed a complete Reibergram did not recognize an inflammatory process and, more crucially, would not have alerted the clinician to possible neurotuberculosis (Albaum and CSF APP). Even worse, 83% of all laboratories (243) that participated in the CSF survey, eventually limited to analyzing the total protein in the CSF, failed to alert the clinician.

The example given in Figure 8 summarizes the three basic aspects of a modern CSF EQAS:

- Analysis of CSF and serum samples in a reliably matched assay procedure (see above).
- Knowledge-based interpretation of the combined data of medically relevant examples.
- CSF and serum samples provided for the survey must enable relevant pattern formation.

With the high IgG, IgA, and IgM concentrations in the CSF, in Figure 8, we obtain a relatively good agreement of the results from all survey participants. However, the interpretation of the IgA quotient requires knowledge-based awareness. Most of the QIgA results given in Figure 8 are below the discrimination limit, QIgA < QIgA(lim), and they were, therefore, interpreted together with the other quotients as barrier dysfunction without inflammatory process. Only 33% of participants recognized that QIgA > QIgG, implying intrathecal IgA synthesis, despite QIgA < QIgA(lim).

The knowledge-based explanation is as follows: the IgA quotient of 64.6 × 10^-3^ was greater than the IgG quotient of 51.6 × 10^-3^. IgA has a larger hydrodynamic radius than IgG and, therefore, must have the smaller quotient due to diffusion (flatter gradient along the diffusion barrier). The normal quotient sequence is QALB > QIgG > QIgA > QIgM. This molecular size-dependent interpretation of the quotients also applies in the case of possible blood contamination, be it artefactual due to puncture or cerebral hemorrhage.

Thus, 67% of the participants did not recognize an inflammatory process and, more importantly, would not have alerted the clinician to a possible case of neurotuberculosis if this was associated with an elevated CSF lactate level (Albaum and Reiber, 2024).

##### 6.3.1.1 The corresponding practical case

We recently learned about the potentially fatal consequences for the patient from a practical case of an IgA analysis in which the very high CSF IgA concentration was beyond the analytical range. The machine in a central laboratory of a university hospital reported the value above the detection limit. The clinical chemist saw no need to measure again with a diluted CSF sample. This would have put her/him in the dilemma of using an assay with a procedure that deviated from the certified protocol. For the patient, this meant a delay in the diagnosis of neurotuberculosis (Figure 3), with 3 wasted weeks of precious treatment.

#### 6.3.2 Samples for CSF testing in the EQAS

The availability of paired CSF and serum samples poses a particular problem in CSF testing.

In practice, residual CSF and serum samples are stored at 4°C after routine analysis and then pooled with different concentration ranges to enable different sample combinations. CSF was rarely obtained from a catheter that had a sufficient volume for all participants in the EQA scheme.

It should be noted that only by using real CSF samples that have an appropriate protein pattern (different protein ratios in CSF and serum due to diffusion through the barrier depending on molecule size) can such a pattern as shown in Figure 8 be created. The use of diluted serum instead of CSF (as practiced by some EQA schemes) does not allow the creation of disease-typical patterns (Albaum and Reiber, 2024). The stereotypical pattern with diluted serum completely deprives the examination of disease-related interpretation training and control.

## 7 Online interpretation software and the certification trap

### 7.1 Online evaluation of protein analysis

The concept of an integrating laboratory report, which presents all laboratory data of a patient together with the interpretation in a quotient diagram (Reiber, 2016c; Wildemann et al., 2010; Reiber, 2016a; Albaum and Reiber, 2024), made it seem obvious to couple the absolute values measured on the nephelometer machine with software that integrates the data directly into an online laboratory data report. These software developments by Albaum (2024) for Dade Behring (now Siemens Healthcare with another software manufacturer) and by Wormek (2024) for Beckman Nephelometers contributed significantly to the acceptance of the Reibergrams. Siemens offers the “Protis” program (Siemens, 2024) as part of an advanced diagnostic concept. However, the expensive accreditation of this long-established software program currently prevents providers from offering their software, e.g., for stand-alone solutions in individual laboratories, possibly in an international context.

### 7.2 Certification and accreditation

Test certification, originally introduced as laboratory marketing, has developed over the decades into a profit-orientated industry in its own right. This has had devastating consequences, particularly for the isolated solutions developed and offered for sale by smaller IT companies. Waiting several years for certification, e.g., by the TÜV (Official technical surveyance of cars in Germany), and, above all, the annual costs of 30,000–50,000 Euros are a deterrent. The additional personnel costs of the software manufacturer for the administrative requirements make, for example, the certification of a simple CSF software program that has been functioning for 30 years prohibitively expensive as these costs cannot be passed on to the user.

The fact that a specialist in metallurgy is sent to the institute for quality assurance in medical diagnostics (INSTAND eV) for accreditation is one of the symptomatic problems of this type of pseudo-inspection. This does not contribute to diagnostic accuracy. Instead, it results in the explosion of costs in the healthcare system. Ultimately, it also means that development in small IT companies is no longer financially viable, and the programs are therefore not even offered. Therefore, this certification practice is an obstruction to quality in medicine.

## 8 Perspectives

The combined data evaluation is a qualified quality control of the laboratory analysis. Qualified, i.e., disease-typical data combinations avoid arbitrary analytical search processes and significantly reduce analysis costs. A purely analytical accuracy testing, as is considered sufficient in most EQA programs, therefore, does not show the necessary qualities of a good laboratory in data processing and interpretation. The misleading trends of assay suppliers must be controlled by medical expertise. Clinical chemists and laboratory physicians are therefore called upon to regain the medical expertise that has been lost through industrial developments. This is the only way to achieve a change in direction from mass analysis to patient-orientated and, at the same time, more cost-effective diagnostics.

The knowledge and responsibility of the clinical chemist cannot be replaced or hindered by certification or accreditation procedures without risk to patients.

## Data Availability

Publicly available datasets were analyzed in this study. These data can be found here: reports to the CSF surveys, www.INSTAND-eV.de.
